# The role of cytokines from salivary gland epithelial cells in the immunopathology of Sjögren’s syndrome

**DOI:** 10.3389/fimmu.2024.1443455

**Published:** 2024-09-13

**Authors:** Yuanji Dong, Ting Wang, Huaxiang Wu

**Affiliations:** ^1^ Department of Rheumatology, The Second Affiliated Hospital of Zhejiang University School of Medicine, Hangzhou, Zhejiang, China; ^2^ Department of Respiratory Disease, Thoracic Disease Center, The First Affiliated Hospital, College of Medicine, Zhejiang University, Hangzhou, Zhejiang, China

**Keywords:** salivary gland epithelial cells, cytokines, Sjögren’s syndrome, lymphoepithelial lesions, tertiary lymphoid structures

## Abstract

In the pathogenesis and progression of Sjögren’s syndrome (SS), hematopoietic cells in the peripheral circulation, tissue-resident immune cells, and parenchymal cells of salivary gland tissues (such as epithelial cells, endothelial cells, fibroblasts, etc.) all play crucial roles. These diverse cells form intricate networks and interact with each other, leading to tissue destruction and persistent chronic inflammation, ultimately causing irreversible damage in glandular function. Among these, salivary gland epithelial cells (SGECs) consistently hold a key position, characterized by their functions in expressing co-stimulatory and antigen-presenting molecules and secreting pro-inflammatory cytokines and chemokines. Moreover, SGECs actively engage in and facilitate the development of specific pathological structures within the salivary gland, such as lymphoepithelial lesions (LELs) and tertiary lymphoid structures (TLSs), thereby substantially elevating the risk of mucosa-associated lymphoid tissue (MALT) lymphoma. Overall, SGECs are recognized for their essential and irreplaceable contributions to the pathogenesis of SS. This review article initially delves into the anatomical composition of salivary gland epithelial cells, subsequently focusing on elucidating the different cytokines derived from SGECs, encompassing chemokines, pro-inflammatory cytokines, anti-inflammatory cytokines, pro-survival cytokines, and damage-associated molecular patterns (DAMPs), to explore their key roles in the pathogenesis of SS.

## 1 Introduction

Sjögren’s syndrome (SS) is a chronic autoimmune disease that typically targets the salivary and lacrimal glands, resulting in xerostomia and xerophthalmia (1). Studies have indicated that the prevalence of SS in adults ranges from 0.2% to 3%, with a female-to-male ratio of 9:1, positioning it as the second most prevalent autoimmune disease following rheumatoid arthritis (2). In terms of clinical presentation, SS is a highly heterogeneous and complex disease, with over 50% of patients experiencing extra-glandular symptoms, including fatigue, interstitial lung disease, interstitial nephritis, peripheral neuropathy, and cryoglobulinemia-associated vasculitis. Approximately 2.7% to 9.8% of SS patients may even develop lymphoma (3). With ongoing research advancements, our understanding of SS has deepened over time. It is currently believed that SS results from a complex interplay of genetic factors, environmental influences (such as microbial infections), and hormonal levels, ultimately leading to disturbances in immune tolerance and abnormal homing of lymphocytes (1, 4). The complex interactions among blood-borne inflammatory cells, tissue-resident immune cells, and parenchymal cells of salivary gland tissues lead to dysfunction of the salivary gland, ultimately resulting in tissue fibrosis. In this process, some unique identifiable structures related to epithelial cells are formed, including LELs (lymphoepithelial lesions) and tertiary lymphatic structures (TLSs) (5, 6). LELs refer to infiltrating lymphocytes (mainly FcRL4+ B cells) within the proliferated striated and excretory ducts. They are highly specific for SS and relatively prevalent, with frequencies reaching up to 56% in the parotid glands and 42% in the labial glands in cohorts of patients with primary Sjögren’s syndrome (pSS) (7). Importantly, LELs are associated with the development of mucosa-associated lymphoid tissue (MALT) lymphoma (6). The TLSs in SS are primarily generated by the interaction of epithelial cells, immune fibroblasts, and infiltrating lymphocytes, which serve as unique and local sites for B cell activation and antibody affinity maturation. It has been reported that approximately 30-40% of SS patients have TLSs in the tissue of the salivary gland. TLSs have also been associated with lymphoma development, although this remains a matter of debate (5, 8–10). Therefore, SGECs hold a special position in the pathogenesis of SS, which is also known as autoimmune epithelitis (11–15).

In addition, epithelial cells, especially ductal epithelial cells, can mimic the functions of antigen-presenting cells by expressing MHC class I and II molecules, as well as co-stimulatory molecules such as CD80, CD86, CD40, and PD-L1. They can also interact with B cells. Studies have shown that SGECs from patients with pSS can significantly promote B lymphocyte activation, which cannot be reversed by inhibitors of individual cytokines (BAFF, APRIL, or IL-6) (16). Furthermore, in LELs, FcRL4+ B cells can directly interact with epithelial cells, leading to B cell expansion, epithelial cell proliferation, and ductal obstruction. In severe cases, this interaction can even induce clonal expansion of B cells, which is a significant driving factor for MALT lymphoma (6, 17–19). Similarly, epithelial cells can also promote T cells to differentiate into Th1 and Tfh cells under the influence of the local inflammatory microenvironment (20, 21). In addition, epithelial cells themselves contain abundant self-antigens and damage-associated molecular patterns. On one hand, SGECs release significant amounts of autoantigens (such as Ro/SSA and La/SSB) through apoptotic blebs and exosomes (22, 23). Additionally, SGECs can secrete BAFF, and chemokines, and upregulate MHC molecules, which are involved in the immune responses of T and B cells, thus promoting the formation of autoantibodies. The antigen-antibody complexes primarily engage Fc receptors (FcR) to activate immune cells and secrete cytokines, which further exacerbate the damage and activation of SGECs. On the other hand, SGECs undergo cell death, including apoptosis, necroptosis, and ferroptosis, in the context of chronic inflammation (24). This process further contributes to the maintenance of the immune microenvironment of SS through the release of DAMPs, such as IL-33 and HMGB1, ultimately leading to impaired glandular function and tissue fibrosis (24). This review article summarizes the cytokines associated with SGECs, including chemokines, pro-inflammatory cytokines, anti-inflammatory cytokines, pro-survival factors, and damage-associated molecular patterns, highlighting their significant and complex roles in SS.

## 2 Anatomy of the salivary glands

**Figure 1:**
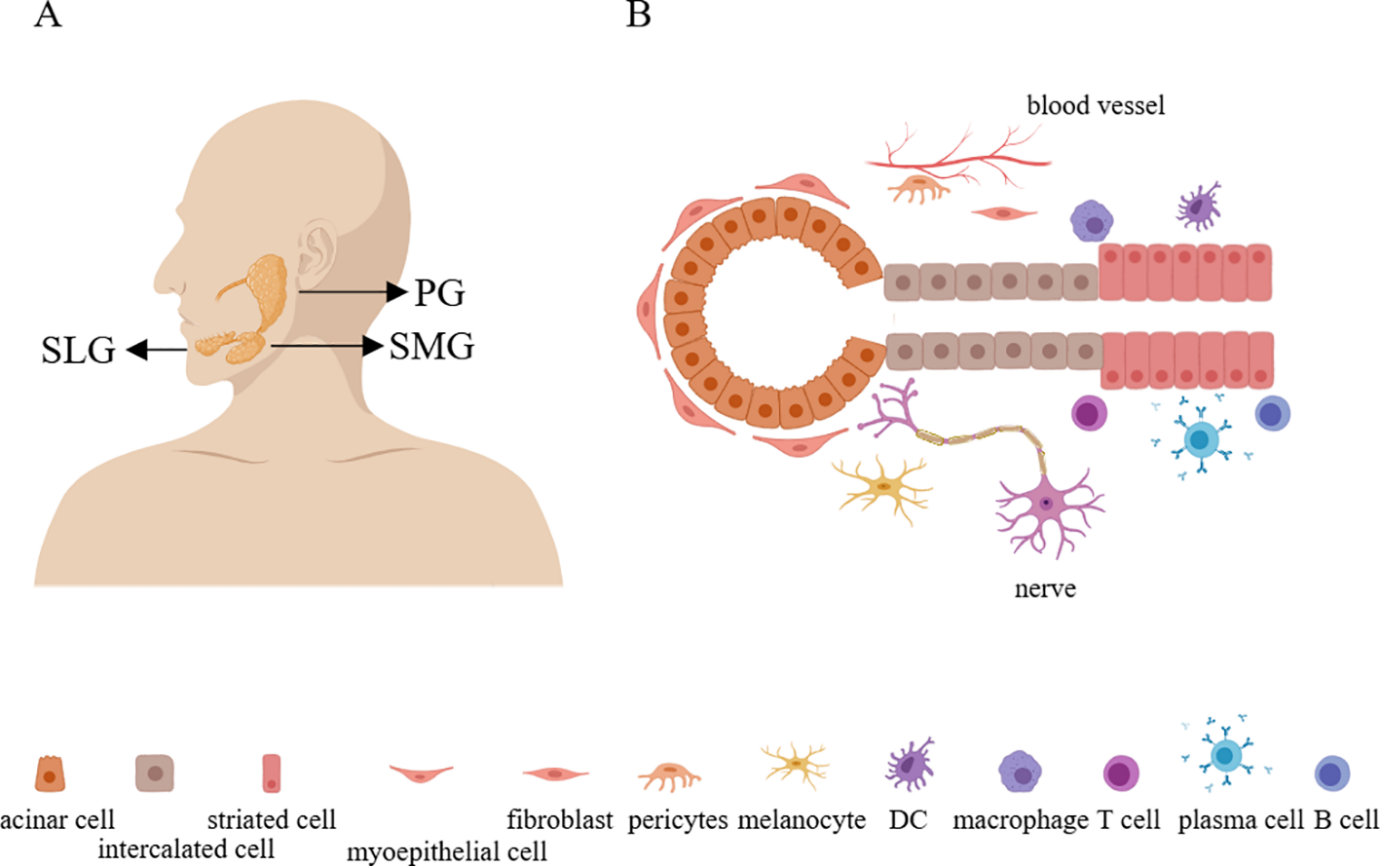
The anatomy and structure of the salivary glands. **(A)** There are three major salivary glands: the parotid gland, the submandibular gland, and the sublingual gland. **(B)** The parenchymal cells of the salivary glands include acinar cells, myoepithelial cells, intercalated duct cells, striated duct cells, melanocytes, fibroblasts, pericytes, and immune cells. Additionally, the salivary glands also contain numerous nerve and blood vessels. PG, parotid gland; SMG, submandibular gland; SLG, sublingual gland.

Salivary glands originate from the epithelial placode during embryonic development and gradually differentiate into mature structures. There are three major salivary glands: the parotid gland (PG), the submandibular gland (SMG), and the sublingual gland (SLG), as well as thousands of minor salivary glands (25) (**Figure 1A**). The secretion ducts of the PG, SMG, and SLG are respectively Stensen’s duct, Wharton’s duct, and Bartholin’s duct. Additionally, the SLG also includes many smaller Rivinus ducts, through which the secreted saliva is emptied into the oral cavity. In humans, the PG is the largest salivary gland, whereas in mice the largest is the SMG (26).

## 3 Composition of salivary gland parenchymal cells

In physiological conditions, salivary glands are primarily composed of epithelial cells, ionocytes (a specialized type of epithelial cell involved in regulating and maintaining osmotic pressure), fibroblasts, smooth muscle cells, endothelial cells, myoepithelial cells, melanocytes, and a small number of immune cells (such as tissue-resident macrophages). Upon continuous interaction with the external environment, more immune cells, including T cells, B cells, plasma cells, and myeloid cells, will accumulate in the salivary gland tissue (25, 27) (**Figure 1B**). Furthermore, there exists a group of cells with differentiation potential in the salivary glands, expressed in both mice and humans, known as salivary gland progenitor cells (SGPCs). These cells can proliferate locally after injury, replenishing lost acinar and ductal tissues. Human SGPCs are located in intercalated ductal, striated ductal, or acinar compartments (28, 29). It is reported that a subset of acinar cells expressing SOX2 can differentiate into MUC19+ acinar cells and play a role in acinar replenishment after radiation-induced damage to the salivary glands (30). Additionally, a subset within the ductal cells expressing the transcription factor ASCL3 can generate ductal cells (31). There is also a population of undifferentiated cells expressing Krt5 within the ductal compartment (26). Moreover, single-cell sequencing and immunofluorescence have shown a significant decrease in SOX9 expression in myoepithelial cells of salivary glands from SS, suggesting the involvement of SOX9 in the transcriptional regulation of myoepithelial cell regeneration (27).

## 4 Salivary gland epithelial cells

SGECs include acinar cells (serous and mucinous acinar cells) which are responsible for secreting saliva (32), myoepithelial cells that support acinar cells and can contract to promote salivary excretion and some of which have differentiation potential (33), intercalated duct cells that may also contribute to salivary secretion (34, 35), and the striate duct cells that dominate salivary transport and regulate ion concentrations (34).

Under the influence of these different epithelial cells, saliva containing water, ions (such as Na+ and Ca2+), immunoglobulins, enzymes, mucins, hormones, and other components ultimately enter the oral cavity. Saliva plays various roles such as aiding in food digestion, cleaning and moisturizing the oral cavity, protecting the mucosa and teeth, providing antimicrobial action, and even promoting wound healing. Therefore, when SGECs are damaged, accompanied by changes in saliva secretion and composition, it significantly affects oral and periodontal health and actively participates in gland inflammation (15, 36, 37). With the establishment of long-term *in vitro* culture systems for SGECs in SS (38), there has been a more in-depth understanding of the functions of these cells, primarily including the functions related to immune regulation and salivary secretion (12, 13). The former primarily involves ductal epithelial cells, as seen in the biopsy tissues of SS patients, where structures resembling TLSs and LELs closely associated with ductal cells are observed (14, 39, 40). Although acinar cells also express some immune-related molecules, they do not seem to recruit or attract immune cells (12). Ductal epithelial cells can recognize pathogen-associated molecular patterns (PAMPs) and damage-associated molecular patterns (DAMPs) through Toll-like receptors (TLRs) (15, 41), which, upon activation, can promote the secretion of type I interferons and B-cell activating factor (BAFF), induce apoptosis, and release self-antigens (42–45). These cells can also express various immune regulatory molecules like ICOSL, CD40, CD80, CD86, MHC molecules, BAFF, CXCL13, and CXCL10, which can influence the survival and differentiation of T and B cells in the local immune microenvironment (12, 13, 21, 46–48). The latter (saliva secretion) mainly involves acinar cells. The secretion of saliva begins with the binding of acetylcholine to M3 receptors (M3R) on acinar cells, leading to an increase in intracellular Ca2+ levels, which further activates aquaporin water channels, such as AQP5, resulting in enhanced salivary secretion. In Sjögren’s syndrome (SS), autoantibodies targeting M3R are produced, and the ability of aquaporin channels to respond to muscarinic stimulation is diminished (49). Additionally, signaling molecules associated with secretion, such as phosphatidylinositol 4,5-bisphosphate (PIP2) and synaptotagmin 1, are mislocalized to the basolateral membrane rather than the apical membrane (50–52). This mislocalization impairs both the production and secretion of saliva, ultimately leading to decreased salivary secretion levels. Furthermore, alterations in mucin distribution and changes in the synthesis and modification of mucins (including decreased glycosylation) also occur, contributing to insufficient saliva lubrication (53, 54). In addition, SGECs from SS are more sensitive to apoptosis than healthy controls (44, 55, 56), whereas ductal cells express the anti-apoptotic protein BCL-2 compared with acinar cells not (57), suggesting that acinar cells are more susceptible to apoptosis. However, local ductal cells may be more prone to damage from inflammatory factors and immune microenvironment (12). Generally speaking, the most pronounced change in acinar cells in SS is the disruption of cellular polarity, particularly affecting proteins related to saliva secretion. Conversely, ductal cells demonstrate more pro-inflammatory features, such as inflammasome activation, NF-κB pathway activation, and cytokine secretion.

## 5 Interaction of salivary gland epithelial cells and immune cells

Local macrophages and dendritic cells in the salivary gland tissue primarily perform immune surveillance functions, particularly in response to infection or damage within the salivary gland. In addition to innate immune cells, CD8+ T cells can be found surrounding the acinar or ductal epithelial cells (58), while CD4+ T cells are mainly located within the ductal epithelial cells (58), and B cells can also infiltrate the ductal epithelial cells (59). These indicate that SGECs are at least spatially close to lymphocytes. In general, tissue-resident CD103+CD8+ T cells play a role in immune surveillance, whereas GZMK+CD8+ T cells primarily exert pro-inflammatory and cytotoxic functions (60, 61). For CD4+ T cells, SGECs can enhance T cell differentiation and proliferation by expressing many immune-related molecules, such as MHC-II, CD80, CD86, CD40, and ICOSL (13, 21). However, despite the theoretical ability of SGECs to present antigens to T cells, there is currently no direct evidence to support this phenomenon. For B cells, SGECs tend to provide a conducive microenvironment. By secreting CXCL10 to attract CXCR3+FcRL4+ B cells, and by secreting BAFF or other yet-to-be-identified factors to promote the survival of intraepithelial B cells (6). These findings suggest that SGECs may interact with T cells by expressing immune-related molecules (MHC-II, CD80, CD86, CD40, ICOSL) and potentially form immune synapses, but further direct evidence is needed to substantiate this phenomenon.

## 6 Cytokines derived from SGECs

SGECs especially ductal epithelial cells can secrete a variety of cytokines, including chemokines, pro-inflammatory cytokines, anti-inflammatory cytokines, and pro-survival cytokines, and DAMPs are actively involved in the pathogenesis of SS (40, 62). Among them, chemokines play a crucial role in recruiting immune cells, pro-inflammatory cytokines coordinate the activation of immune cells, anti-inflammatory cytokines participate in local immune regulation, pro-survival cytokines assist in the survival and differentiation of immune cells, and damage-associated molecular patterns act as a bridge between innate and adaptive immunity. The following section summarizes the roles of cytokines derived from SGECs in SS (**Table 1**).

**Table 1:** The composition and function of SGECs-derived cytokines in SS.

Cytokines	Related molecules	Function	LELs related molecules	TLSs related molecules	Refs
Chemokines	CCL2, CCL3, CCL4, CCL5, CCL6, CCL11, CCL19, CCL20, CCL26, CXCL1, CXCL2, CX- CL8, CXCL10, CXCL12, CXCL13, CX3CL1	recruiting inflammatory cells	CXCL10	CCL19, CXCL12, CXCL13	(11, 12, 14, 65–76)
Pro-inflammatory cytokines	IL-1β, IL-6, IL-18, IL-25, IL-2, TNF-a, IFN-β, IFN- lambda, IL-13, IL-15, GM-CSF	activation of the immune cells	IL-6, IFN-β	IL-13	(11, 12, 14, 77–79, 83–90)
Anti-inflammatory cytokines	TGF-β, TSLP, IL-37. IL- 10	maintenance tolerance, suppression of immune cells	NA	NA	(84, 92–97)
Pro-survival cytokines	BAFF, IL-7	maintaining of immune cell survival	BAFF	BAFF, IL- 7	(46, 47, 85, 86, 98)
DAMPs	HMGB1, IL-1α, IL-33	connecting in- nate and adap- tive immunity	NA	NA	(19, 119–127)

SGECs, salivary gland epithelial cells; SS, Sjögren's Syndrome; LELs, lymphoepithelial lesions; TLSs, tertiary lymphoid structures; CCL2, C-C motif chemokine ligand 2; CXCL1, C-X-C motif chemokine ligand 1; CX3CL1, C-X3-C motif ligand 1; IL-1β, interleukin-1β; TSLP, thymic stromal lymphopoietin; TNF-a, tumor necrosis factor-a; IFN-β, interferonß; GM- CSF, granulocyte-macrophage colony-stimulating factor; TGF-ß, transforming growth factor; BAFF, B-cell Activating factor of the TNF family; HMGB1, high mobility group protein B1; NA, not applicable; Refs, references.

### 6.1 Chemokines derived from SGECs

**Figure 2:**
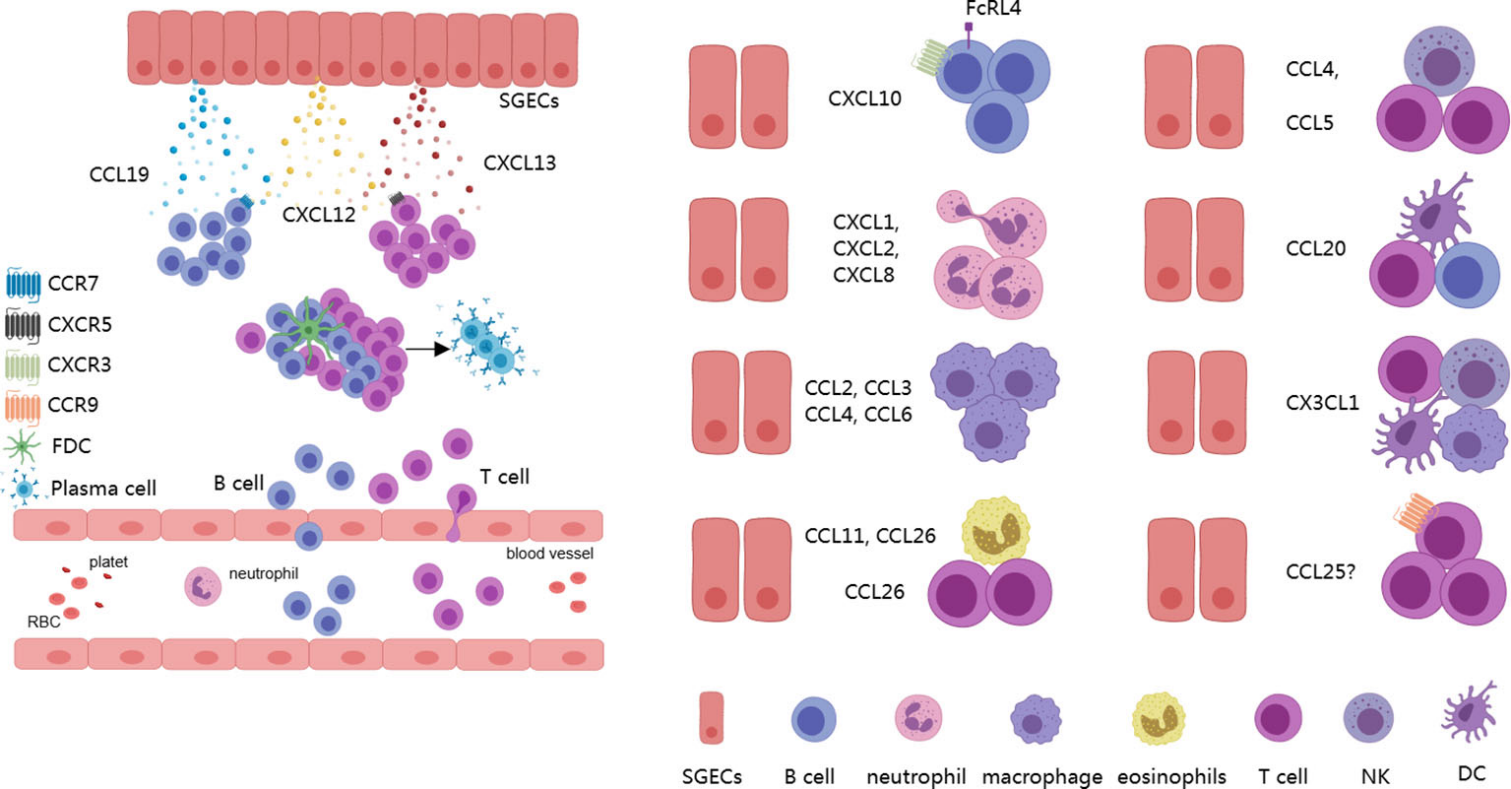
Chemokines derived from SGECs. SGECs can secrete various chemokines to recruit different immune cells, including B cells, T cells, NK cells, as well as myeloid lineage-related cells, which contribute to the formation of TLSs and LELs, actively participating in the progression of the disease. SGECs, salivary gland epithelial cells; TLSs, tertiary lymphoid structures; LELs, lymphoepithelial lesions.

Chemokines and their receptors are important communication tools for interactions between different cells (63), serving as a key link in understanding the formation of the complex pathological microenvironment in SS (62). Currently, over 50 chemokines have been identified in both mice and humans, which can be classified into subfamilies based on differences in the N-terminal cysteine motif, including the CC, CXC, XC, and CX3C chemokine subfamilies. Correspondingly, there are 18 receptors categorized into the CC, CXC, XC, and CX3C chemokine receptor subfamilies (64, 65). SGECs can secrete a variety of chemokines in different inflammatory microenvironments, including CCL3, CCL4, CCL5, CCL11, CCL19, CXCL8, CXCL10, CXCL12, CXCL13, and CX3CL1 (13, 15, 66–70). Additionally, in senescent (P16+) cells, further secretion of CCL2, CCL6, CCL20, CCL26, CXCL1, and CXCL2 has been reported (12, 71). These chemokines can bind to various chemokine receptors and recruit inflammatory cells. CCL2, CCL3, CCL4, and CCL6 exhibit chemotactic activities towards monocytes/macrophages, while CCL4 can also attract NK cells. CCL5 attracts T lymphocytes, CCL11 attracts eosinophils, and CCL19 primarily recruits naïve T cells, central memory T cells, regulatory T cells, naïve B cells, dendritic cells, and NK cells. CCL20 can recruit dendritic cells, Th17 cells, and B cells, while CCL26 attracts eosinophils and Th2 cells. CXCL1, CXCL2, and CXCL8 are mainly responsible for attracting neutrophils, CXCL10 primarily attracts Th1 cells and CXCR3+ B cells, and CXCL12 can attract T cells and monocytes. CXCL13 predominantly attracts CXCR5+ B cells. CX3CL1 attracts T cells, NKT cells, monocytes/macrophages, and dendritic cells, and it promotes the progression of SS-like phenotypes in NFS/sld mice after thymectomy (70, 72, 73). Additionally, CCL25 may be expressed in SGECs, which primarily attract CCR9+ T cells (74). Current evidence supports an increase in protein levels of CCL25 in salivary gland tissues, with CCR9+ T cells observed near SGECs. In other mucosal sites, such as the intestinal tract, epithelial cells are known to express high levels of CCL2 (74–76). Furthermore, CCL19, CXCL12, and CXCL13 are associated with the formation of TLSs (5). CXCL10 is closely related to the formation of LELs and can recruit CXCR3+FcRL4+ memory B cells (6, 12, 77) (**Figure 2**). These data suggest that, under the promotion of senescent and inflammatory phenotypes, the chemokines secreted by SGECs weave into a network, greatly accelerating the progression of SS.

### 6.2 Pro-inflammatory cytokines derived from SGECs

Although SGECs produce cytokines to a lesser extent compared to immune cells, they are ubiquitous in salivary glands and often encounter pathogens earlier than immune cells. To effectively protect the body from pathogen damage, SGECs can secrete pro-inflammatory cytokines to coordinate the activation of immune cells recruited to the local area. SGECs can secrete a variety of pro-inflammatory cytokines, including IL-1β, IL-6 (13), IL-8 (CXCL8), IL-18, IL-25, TSLP, IL-2, TNF-a, IFN-β, IFN-lambda (type III interferon), and IL-13. Additionally, senescent cells can also produce IL-15 and GM-CSF (12, 13, 15, 78–80). Viral infection is one of the significant triggers for the production of cytokines by salivary gland epithelial cells in SS, which is commonly observed in patients and plays a role in disease progression, such as by the elevation of type I interferons or molecular mimicry (81). Among the viruses studied in the context of SS, Epstein-Barr virus (EBV) and human T-cell leukemia virus type 1 (HTLV-1) have garnered considerable attention (81). Interestingly, EBV primarily infects B cells during asymptomatic carriage or chronic infection, however, upon reactivation of the virus (for example, through the use of aryl hydrocarbon receptor (AhR) agonists et.), EBV can also infect SGECs, serving as units for viral replication (82, 83). This can lead to the activation of endogenous toll-like receptors (TLRs), resulting in the release of inflammatory cytokines and type I interferon, or it may ultimately cause cell lysis through cytotoxic effects of immune cells, thereby releasing damage-associated molecular patterns (DAMPs). HTLV-1 appears to influence the functional activities of SGECs through direct infection, and it has been shown to increase the production of pro-inflammatory factors such as intercellular cell adhesion molecule-1 (ICAM-1) (81).

**Figure 3:**
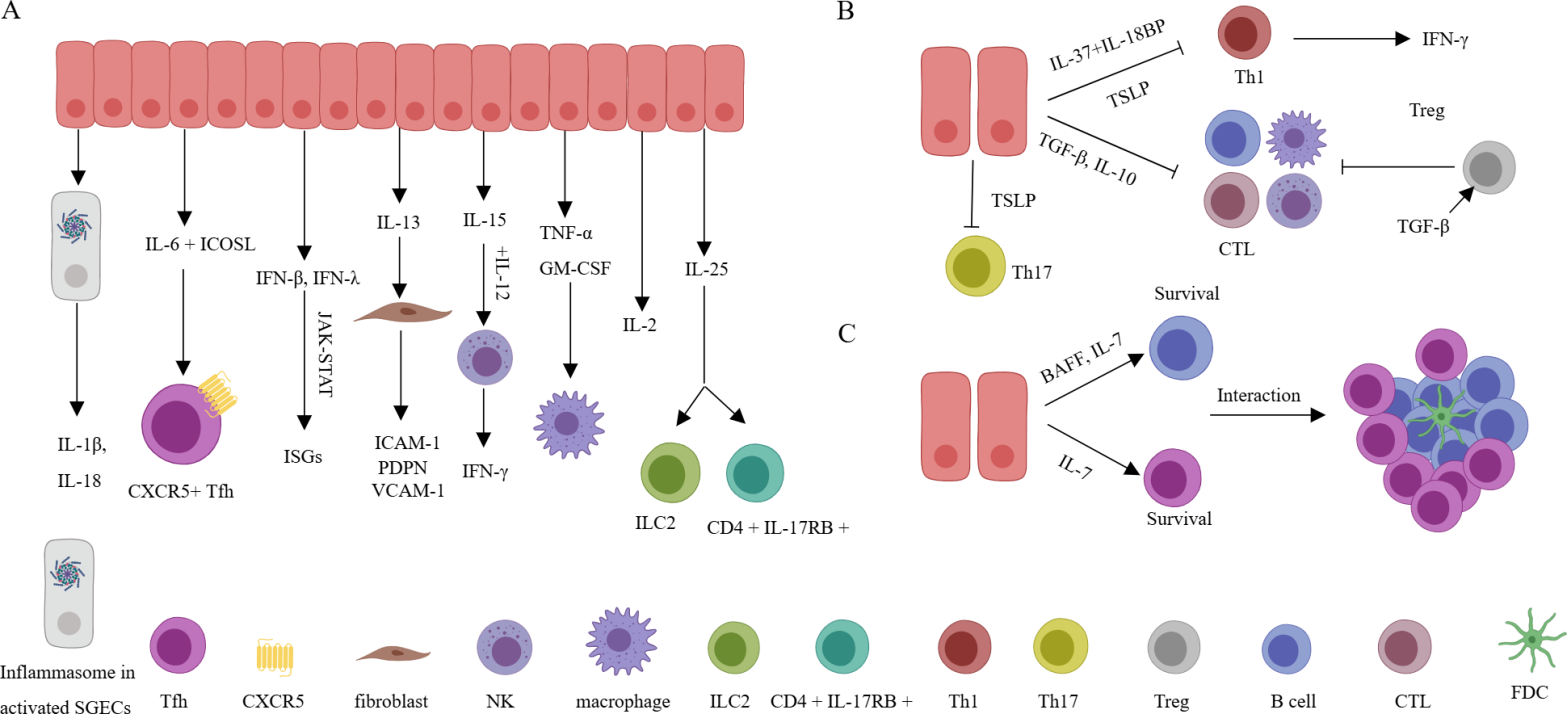
Inflammatory cytokines, anti-inflammatory cytokines, and pro-survival cytokines derived from SGECs. **(A)** Various inflammatory factors are actively involved in the activation of immune cells or stromal cells, leading to the production of more inflammatory factors, and resulting in a sustained chronic inflammatory environment. **(B)** Through anti-inflammatory factors, excessive inflammation damage is avoided, but the downregulation of anti-inflammatory factor expression indirectly promotes and maintains the inflammatory microenvironment, exacerbating disease progression. **(C)** By secreting pro-survival factors, the long-term survival of local immune cells is maintained, promoting the formation of structures related to TLSs and LELs. SGECs, salivary gland epithelial cells; TLSs, tertiary lymphoid structures; LELs, lymphoepithelial lesions; FDC, follicular dendritic cells; CTL, Cytotoxic T lymphocytes.

Activated SGECs can spontaneously secrete IL-1β, a process related to the accumulation of cytoplasmic DNA in SGECs and activation of the AIM2 inflammasome. IL-1β actively participates in cell proliferation, differentiation, and apoptosis and drives the progression of immunity and inflammation (78, 84). SGECs can produce IL-6 when stimulated synergistically by IL-17 and IL-18. IL-6 then promotes B cell proliferation and, in coordination with ICOSL expressed by SGECs, facilitates the differentiation of T cells into T follicular helper cells (Tfh) (21, 80). Furthermore, it has been determined that both acinar cells and ductal cells express IL-18 by immunohistochemistry. Additionally, *in vitro* studies have shown that human SGEC lines HSY and AZA3 cells can be induced to secrete IL-18. IL-18, in turn, can induce Th1 cells to secrete IFN-γ (80). In addition, immunohistochemistry has also demonstrated the expression of IL-25 in SGECs surrounding infiltrating CD4+ T cells. Extracellular IL-25 can promote the expansion of inflammatory ILC2, increase the concentration of autoantibodies, and enhance the frequency of CD4+ IL-17RB+ TRAF6+ cells, contributing to the pathogenesis of SS and SS-related lymphoma (79). It has been reported that SGECs can also express IL-2 and TNF-α, which has been validated at the transcriptional level, however, the actual significance still requires further research (85). Stimulation of TLR3 of SGECs by Poly I: C can lead to the secretion of IFN-β. This, in turn, through the JAK-STAT pathway, influences cell proliferation and regulates immune responses. Additionally, under TLR3 stimulation, SGECs also express interferon-lambda, which plays a role in the production of BAFF and CXCL10 (86, 87). In the resting state, SGECs can secrete IL-13, which can stimulate IL-13R+ fibroblasts to express VCAM-1, PDPN, and ICAM-1, which is related to form TLSs (88). Senescent cells or SGECs activation by TLR2 can produce IL-15, which can induce the proliferation of B cells, T cells, and NK cells. It can also collaborate with IL-12 to stimulate NK cells to produce IFN-γ, contributing to inflammation in the SS microenvironment (89, 90). Senescent cells can also produce GM-CSF, which can participate in the immune response by increasing neutrophils and monocytes and enhancing the phagocytic action of macrophages (12, 91) (**Figure 3A**). These data indicate that the proinflammatory cytokines secreted by SGECs play a significant role in SS, contributing to the supplementation and maintenance of local mucosal immune function. However, persistent chronic inflammation can lead to tissue damage and the generation of TLSs. Targeting proinflammatory cytokines and inflammatory pathways to restore normal epithelial cell function holds therapeutic significance for SS.

### 6.3 Anti-inflammatory cytokines derived from SGECs

In addition to regulating the secretion of saliva components, SGECs, functioning as barrier cells, also secrete various anti-inflammatory cytokines. Despite not being regularly exposed to harmless antigens (e.g. food) like the intestines, salivary glands are also open to the oral cavity, directly interacting with the external environment. The oral cavity hosts a diverse microbial community and is prone to viral infiltration. This underscores the importance of salivary gland tissues maintaining sufficient tolerance to normal oral microbiota while responding actively to harmful pathogens. Intestinal epithelial cells can constitutively express TGF-β, helping to keep resident immune cells relatively quiescent (92). Similarly, SGECs also express TGF-β, which might contribute to local microenvironment tolerance (93). At the same time, TGF-β also participates in the fibrosis of the salivary gland (94). Although TGF-β can promote the differentiation of Th17 or Th9 cells in specific conditions where IL-6 or IL-4 are present, exerting pro-inflammatory effects, generally, TGF-β plays an anti-inflammatory effect by promoting Treg cells and inhibiting cytotoxic and Th1 cells (95). Similarly, TSLP also has complex pro-inflammatory and anti-inflammatory effects, playing important roles in allergic reactions and antiviral immunity (96). In SS, the reduced expression of TSLP in SGECs may contribute to the increase of Th1 and Th17 cells, thereby promoting the progression of SS (97). IL-37 is an inhibitor of gene expression and inflammation located within the cell nucleus. It can bind to the IL-18 receptor (IL-18R), leading to the inhibition of transcription factors NF-κB and MAPK. Additionally, IL-37 can interact with IL-18 binding protein (IL-18BP), a natural inhibitor of IL-18-mediated inflammatory activity, to suppress the synthesis of IFN-γ (98). Additionally, SGECs also express IL-10, which exerts significant inhibitory effects on both T and B cells (85) (**Figure 3B**). SGECs concurrently express anti-inflammatory cytokines, which help maintain homeostasis and play a therapeutic role in potential tissue damage. The imbalance between anti-inflammatory and pro-inflammatory cytokines leads to the ongoing progression of SS.

### 6.4 Pro-survival cytokines derived from SGECs

SGECs also simultaneously express cytokines that promote the survival of infiltrating T and B cells. Various cytokines, such as Type I interferons and interferon-lambda, can enhance ductal cell production of BAFF (48, 87, 99). BAFF is essential for B cell survival. BAFF also guides B cell maturation, development, survival, as well as immunoglobulin production and class switching (100–102). BAFF also enhances B cell’s (especially memory B cells) chemotactic response to CXCL13 (103). Although BAFF plays a crucial role in the survival and activation of local B cells, inhibiting BAFF alone in the interaction between SGECs isolated from SS patients and B cells *in vitro* did not affect B cell survival. This suggests other factors under these experimental conditions can also influence B cell survival (16). In addition, transcriptional analysis of SGECs from both SS and sicca patients showed a significant increase in IL-7 expression. Furthermore, treatment with Poly (I: C), type I interferons, IFN-γ, or interferon-lambda can all stimulate the secretion of IL-7 by SGECs (47, 86). Interestingly, IL-7 not only contributes to the development of B cells and the generation and survival of T cells (104, 105), but it can also activate Th1 cells to produce IFN-γ, indirectly promoting the expression of CXCL10 (106). Additionally, IL-7 assists in activating CCR9+ T cells to produce IFN-γ, IL-17, and IL-21 (107). IL-7 is also related to the formation of TLSs (5, 108) (**Figure 3C**). These data suggest that both BAFF and IL-7 can significantly promote the survival of infiltrating lymphocytes and contribute to the maintenance and development of the local inflammatory microenvironment.

### 6.5 The death patterns of SGECs

**Figure 4:**
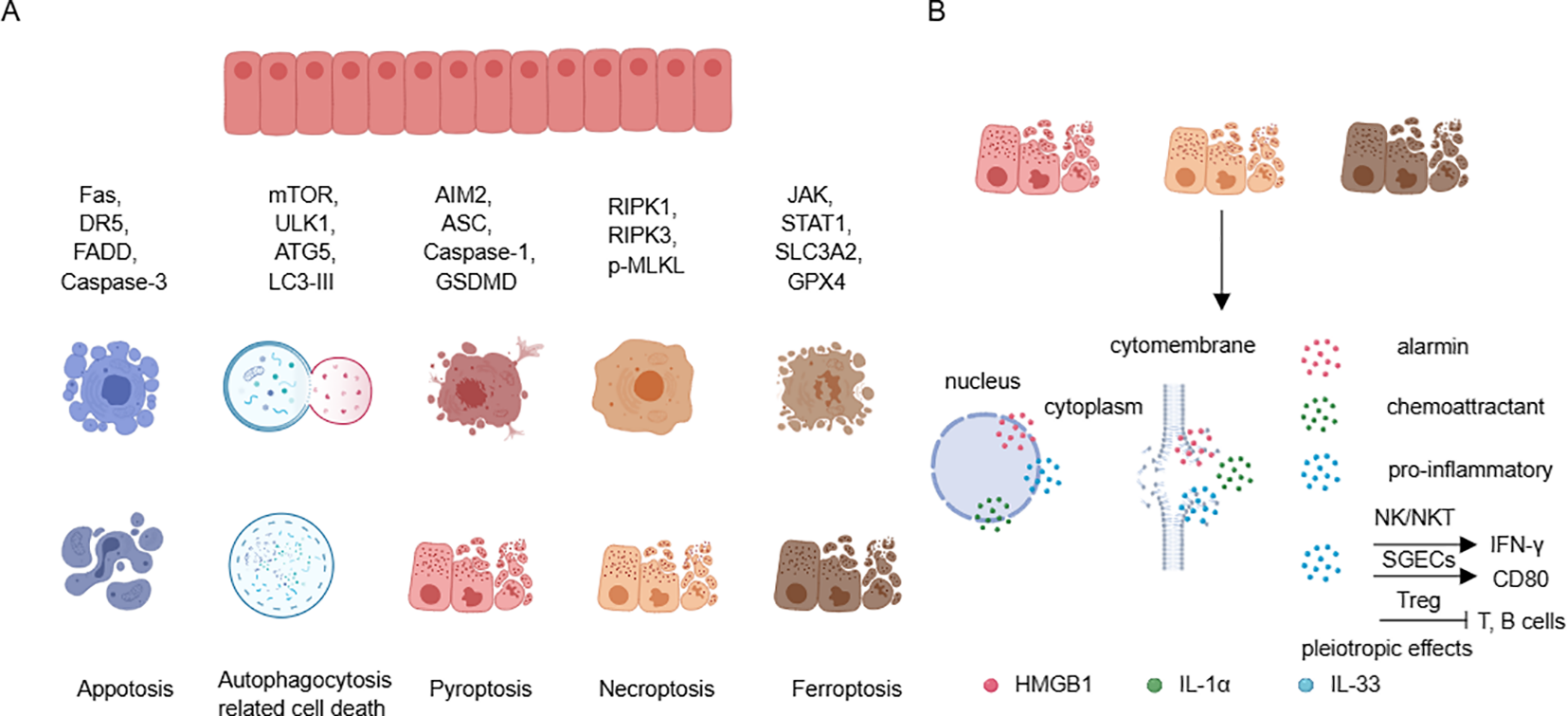
Cell death in SGECs and releasing of DAMPs. **(A)** SGECs can participate in various forms of cell death by recruiting different molecules. **(B)** Cell death, especially pyroptosis, necroptosis, and ferroptosis, can lead to the release of HMGB1, IL-1α, and IL-33, promoting and maintaining local inflammation, with IL-33 exhibiting pleiotropic effects. SGECs, salivary gland epithelial cells; DAMPs, damage-associated molecular patterns.

SGECs are also one of the main sources of local alarmins, especially during the process of inflammatory cell death (109). It has been reported that SGECs can undergo various forms of cell death (24). Apoptosis is the most common form of cell death in salivary SGECs in SS, involving both extrinsic apoptosis pathways induced by Fas/FasL and TRAIL, as well as intrinsic apoptosis pathways induced by mitochondria (110). While traditional views consider apoptosis as a non-inflammatory form of cell death, impairments in the phagocytic process can lead to secondary necrosis associated with inflammation. Additionally, the formation of apoptotic bodies may result in the loss of immune tolerance, leading to the production of autoantibodies (111). Autophagy also actively contributes to the pathogenesis of SS. It has been demonstrated that there is excessive autophagy in SGECs of SS patients, exacerbating the inflammatory response (112, 113), in contrast, in the MRL/lpr mice (mimic SS model), insufficient autophagy is observed, leading to reduced clearance of dead cells and increased autoantigens (114). These differences may be related to the stage of disease onset and the local inflammatory microenvironment. Furthermore, in the immune microenvironment of SS, SGECs exhibit damaged DNAse1, leading to abnormal accumulation of cytoplasmic DNA and excessive activation of AIM2. Moreover, after Type I interferon stimulation, SGECs can undergo pyroptosis associated with NLRP3 or AIM2 inflammasome activation (84, 115). Other inflammatory cytokines like IL-17 can stimulate SGECs to express high levels of pMLKL and RIPK3 (116). Additionally, IFN-γ can downregulate solute carrier family 3 member 2 (SLC3A2), glutathione, and GPX4 through the JAK/STAT1 pathway, thereby triggering ferroptosis in SGECs (117) (**Figure 4A**). These findings suggest that in the SS microenvironment, salivary gland epithelial cells may undergo necroptosis and ferroptosis (116, 118). These modes of cell death enable DAMPs to be released from SGECs and exert significant pro-inflammatory effects.

### 6.6 DAMPs derived from SGECs

This review article focuses on the DAMPs including HMGB1, IL-1α, and IL-33, Under normal circumstances, these cytokines are located in the cell nucleus. HMGB1, in particular, can be actively released under certain stimuli, as well as passively released during inflammatory cell death processes (119). The active release of HMGB1 is partly facilitated by a JAK/STAT1 signaling-mediated acetylation of lysine residues which promotes its translocation from the nucleus to the cytoplasm, and further secretion into the extracellular space through the secretory lysosome pathway (120). In contrast, the passive release of HMGB1 mainly involves the inflammatory cell death processes, which release the reduced (primarily chemoattractive) and disulfide isoforms (primarily pro-inflammatory) (121, 122). The SGECs can undergo inflammatory cell death which provides a rich source of HMGB1. Moreover, elevated levels of HMGB1 have been found in the peripheral blood of SS patients, correlating with extra-glandular involvement (123). Furthermore, HMGB1-neutralizing antibodies are believed to significantly alleviate the inflammatory phenotype of SS (124). IL-1α is another nuclear factor, and compared with healthy control, there is an increase in IL-1α transcripts in SGECs of SS. Additionally, the staining intensity of IL-1α is increased in the salivary gland ducts and acinar cells of SS. After inflammatory cell death, IL-1α is released into the surrounding environment as an alarmin, and it activates downstream pathways in an IL-1R1-dependent manner to promote the recruitment of chemokines and inflammatory cells (125). IL-33 is a dual-functional nuclear protein that can activate both type 1 and type 2 immune cells in an environmentally specific manner through its receptor ST2 (126). Recent studies have revealed a type 1 immune-restricted promoter for the ST2 encoding gene Il1rl1 that is crucial for antiviral CD8+ cytotoxic T cell and CD4+ Th1 cell responses. Interestingly, ST2 is only transiently expressed post-activation under the drive of T-bet and STAT4, in contrast to the high basal level constitutive expression of ST2 under the GATA-3 drive (127). In SS, IL-33 is expressed in acinar, ductal, and endothelial cells, and is released in damaged tissues during inflammatory infiltration, which is in agreement with that IL-33 is increased in tissues with Chisholm scores of 2 or 3 and decreased in tissues with Chisholm scores of 4, both indicating that IL-33 can be released to the extracellular to exert its function (20, 128). In addition, IL-33 is thought to synergize with IL-12 and IL-23 to promote IFN-γ production by NK and NKT cells (128). One of our studies found that IL-33 was able to promote the phenotype of APC in salivary gland epithelial cells and contribute to the differentiation of T cells in the Th1 (20) (**Figure 4B**). Taken together, these studies suggest that SGECs-derived DAMPs can actively participate in the pathology of SS, thus maintaining normal epithelial function and reducing inflammatory death, which are effective therapeutic targets.

## 7 Therapies targeting SGECs

SGECs play a complex and crucial role in the progression of SS. Therefore, reshaping the function of SGECs requires not only altering the local immune microenvironment but also improving the senescent phenotype, reducing inflammatory cell death of SGECs, and replenishing with newly regenerated parenchymal cells. Currently, there is a lack of specific treatments targeting SGECs. Ongoing therapies include but are not limited to, SYK inhibitors (NCT03100942), BTK kinase inhibitors (NCT03100942), baricitinib (NCT04916756, NCT05016297), tofacitinib (NCT04496960, NCT05087589), BAFF inhibitors (NCT05349214, NCT05350072, NCT01160666), and rapamycin (NCT05605665). Some studies have shown positive results in the treatment of SS, such as ianalumab (VAY736) (129), baricitinib (130), and tofacitinib (131), but the assessment of epithelial cell benefits is insufficient due to a lack of relevant evaluation metrics. In the future, it is essential to develop more indicators related to the improvement of epithelial cell function. Other treatments include senescent cell exhaustion therapy (132, 133) and stem cell therapy, where patient-matched pluripotent stem cells are differentiated into SGECs and transplanted back into the patient (26, 134, 135). However, long-term follow-up is necessary to monitor functional restoration and tumor risks. With the ongoing advancements in monoclonal antibody drugs, small molecule drugs, stem cell regenerative technology, and organoid research, it is believed that there will be more effective methods in the future for treating SS and reshaping the tissue microenvironment of salivary glands.

## 8 Discussion

**Figure 5:**
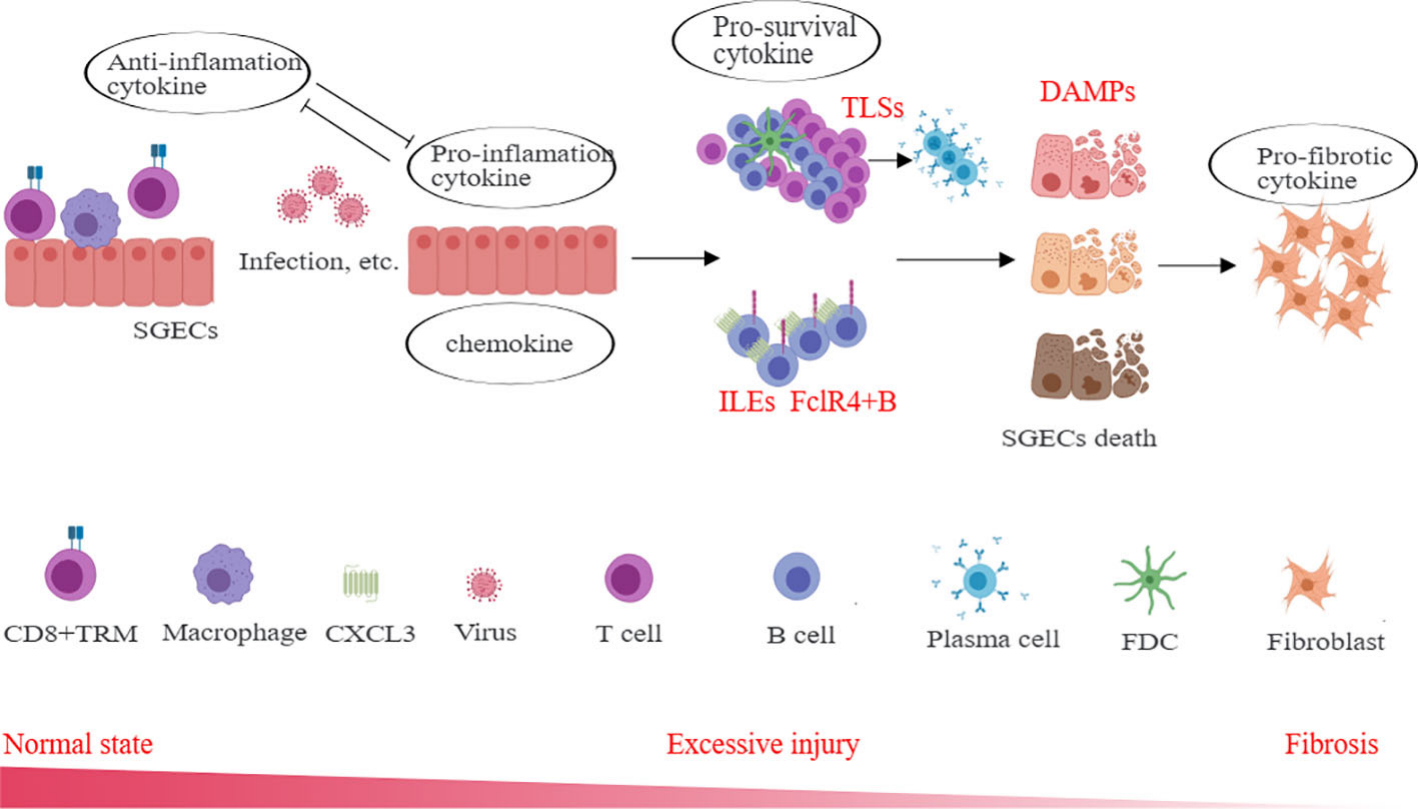
The role of cytokines derived from SGECs in SS immunopathogenesis. Different cytokines including chemokines, pro-inflammatory cytokines, anti-inflammatory cytokines, pro-survival cytokines, and DAMPs et. actively participate in the inflammation, and through indirect or direct interactions, lead to excessive damage and fibrosis of the salivary gland. SGECs, salivary gland epithelial cells; TLSs, tertiary lymphoid structures; LELs, lymphoepithelial lesions; DAMPs, damage-associated molecular patterns; TRM, tissue-resident memory T cells; FDC, follicular dendritic cells.

SGECs play a crucial role in the pathogenesis of SS and are associated with important pathological structures in SS, including TLSs and LELs. One of the most important roles of SGECs is the APC phenotype, allowing them to secrete various cytokines, including chemokines, pro-inflammatory cytokines, anti-inflammatory cytokines, pro-survival cytokines, and DAMPs. Chemokines play a role in recruiting inflammatory cells from circulation, which is associated with the decreased levels of certain subgroups found in the peripheral blood of SS patients. Pro-inflammatory cytokines can maintain the activation of inflammatory cells, exacerbate tissue damage, and inhibit the function of anti-inflammatory cytokines. Pro-survival cytokines can sustain the long-term survival of local inflammatory cells, especially lymphocytes, promoting interactions between lymphocytes and parenchymal cells, ultimately leading to inflammatory cell death of SGECs, which results in the release of DAMPs, further promoting the release of chemokines and pro-inflammatory factors, leading to chronic tissue inflammation, tissue damage, gland dysfunction, and tissue fibrosis (**Figure 5**). This suggests that timely rescue of damaged epithelial cells and restoration of their normal function are of significant therapeutic importance in SS. Currently, there is a limited number of drugs targeting SGECs, highlighting the need for a deeper understanding of the functions of these cells, including metabolism and specific immune characteristics, which may be one of the key directions for future SS treatments.

## 9 Conclusion

SS is a chronic inflammatory autoimmune disease, and SGECs play an irreplaceable role in the progression of SS. Understanding the function of SGECs is crucial for comprehending the pathophysiology of the disease and developing therapeutic targets. We summarize the cytokines produced by SGECs, highlighting the importance of restoring their function and rescuing damaged epithelial cells. In the future, it will be essential to develop new targets for inhibiting the activation of SGECs and preventing the inflammatory death of SGECs to provide innovative directions for the treatment of SS.

## References

[1] Longhino S Chatzis LG Dal Pozzolo R Peretti S Fulvio G La Rocca G. Sjögren’s syndrome: one year in review 2023. Clin Exp Rheumatol. (2023) 41:2343–56. doi: 10.55563/clinexprheumatol/255qsx 38149515

[2] Qin B Wang J Yang Z Yang M Ma N Huang F. Epidemiology of primary Sjögren’s syndrome: a systematic review and meta-analysis. Ann Rheum Dis. (2015) 74:1983–9. doi: 10.1136/annrheumdis-2014-205375 24938285

[3] Nocturne G Pontarini E Bombardieri M Mariette X. Lymphomas complicating primary Sjögren’s syndrome: from autoimmunity to lymphoma. Rheumatol (Oxford). (2021) 60:3513–21. doi: 10.1093/rheumatology/kez052 PMC8328496 30838413

[4] Mariette X Criswell LA. Primary sjögren’s syndrome. N Engl J Med. (2018) 378:931–9. doi: 10.1056/NEJMcp1702514 29514034

[5] Dong Y Wang T Wu H. Tertiary lymphoid structures in autoimmune diseases. Front Immunol. (2023) 14:1322035. doi: 10.3389/fimmu.2023.1322035 38259436 PMC10800951

[6] Pringle S Verstappen GM van Ginkel MS Nakshbandi U Girigoria Z Bootsma H. Lymphoepithelial lesions in the salivary glands of primary Sjögren’s syndrome patients: the perfect storm? Clin Exp Rheumatol. (2022) 40:2434–42. doi: 10.55563/clinexprheumatol/06an99 36226606

[7] van Ginkel MS van der Sluis T Bulthuis MLC Buikema HJ Haacke EA Arends S. Digital image analysis of intraepithelial B-lymphocytes to assess lymphoepithelial lesions in salivary glands of Sjögren’s syndrome patients. Rheumatol (Oxford). (2022) 62:428–38. doi: 10.1093/rheumatology/keac212 PMC9788820 35412585

[8] Theander E Vasaitis L Baecklund E Nordmark G Warfvinge G Liedholm R. Lymphoid organisation in labial salivary gland biopsies is a possible predictor for the development of Malignant lymphoma in primary Sjögren’s syndrome. Ann Rheum Dis. (2011) 70:1363–8. doi: 10.1136/ard.2010.144782 PMC3128323 21715359

[9] Sène D Ismael S Forien M Charlotte F Kaci R Cacoub P. Ectopic germinal center-like structures in minor salivary gland biopsy tissue predict lymphoma occurrence in patients with primary sjögren’s syndrome. Arthritis Rheumatol. (2018) 70:1481–8. doi: 10.1002/art.40528 29669392

[10] Haacke EA van der Vegt B Vissink A Spijkervet FKL Bootsma H Kroese FGM. Germinal centres in diagnostic labial gland biopsies of patients with primary Sjögren’s syndrome are not predictive for parotid MALT lymphoma development. Ann Rheum Dis. (2017) 76:1781–4. doi: 10.1136/annrheumdis-2017-211290 28710097

[11] Tang Y Zhou Y Wang X Che N Tian J Man K. The role of epithelial cells in the immunopathogenesis of Sjögren’s syndrome. J Leukoc Biol. (2024) 115:57–67. doi: 10.1093/jleuko/qiad049 37134025

[12] Verstappen GM Pringle S Bootsma H Kroese FGM. Epithelial-immune cell interplay in primary Sjögren syndrome salivary gland pathogenesis. Nat Rev Rheumatol. (2021) 17:333–48. doi: 10.1038/s41584-021-00605-2 PMC8081003 33911236

[13] Manoussakis MN Kapsogeorgou EK. The role of intrinsic epithelial activation in the pathogenesis of Sjögren’s syndrome. J Autoimmun. (2010) 35:219–24. doi: 10.1016/j.jaut.2010.06.011 20685080

[14] Wang X Bootsma H de Koning J Kroese FGM Pringle S. Novel approaches for rescuing function of the salivary gland epithelium in primary Sjögren’s syndrome. Clin Exp Rheumatol. (2020) 38 Suppl 126:261–70. 33095136

[15] Goules AV Kapsogeorgou EK Tzioufas AG. Insight into pathogenesis of Sjögren’s syndrome: Dissection on autoimmune infiltrates and epithelial cells. Clin Immunol. (2017) 182:30–40. doi: 10.1016/j.clim.2017.03.007 28330683

[16] Rivière E Pascaud J Tchitchek N Boudaoud S Paoletti A Ly B. Salivary gland epithelial cells from patients with Sjögren’s syndrome induce B-lymphocyte survival and activation. Ann Rheum Dis. (2020) 79:1468–77. doi: 10.1136/annrheumdis-2019-216588 32843324

[17] Haacke EA Bootsma H Spijkervet FKL Visser A Vissink A Kluin PM. FcRL4(+) B-cells in salivary glands of primary Sjögren’s syndrome patients. J Autoimmun. (2017) 81:90–8. doi: 10.1016/j.jaut.2017.03.012 28390747

[18] Visser A Verstappen GM van der Vegt B Vissink A Bende RJ Bootsma H. Repertoire analysis of B-cells located in striated ducts of salivary glands of patients with sjögren’s syndrome. Front Immunol. (2020) 11:1486. doi: 10.3389/fimmu.2020.01486 32760405 PMC7372116

[19] Verstappen GM Ice JA Bootsma H Pringle S Haacke EA de Lange K. Gene expression profiling of epithelium-associated FcRL4(+) B cells in primary Sjögren’s syndrome reveals a pathogenic signature. J Autoimmun. (2020) 109:102439. doi: 10.1016/j.jaut.2020.102439 32201227 PMC7337041

[20] Dong Y Ming B Gao R Mo Q Wu X Zheng F. The IL-33/ST2 axis promotes primary sjögren’s syndrome by enhancing salivary epithelial cell activation and type 1 immune response. J Immunol. (2022) 208:2652–62. doi: 10.4049/jimmunol.2101070 35649629

[21] Gong YZ Nititham J Taylor K Miceli-Richard C Sordet C Wachsmann D. Differentiation of follicular helper T cells by salivary gland epithelial cells in primary Sjögren’s syndrome. J Autoimmun. (2014) 51:57–66. doi: 10.1016/j.jaut.2013.11.003 24411167

[22] Kapsogeorgou EK Abu-Helu RF Moutsopoulos HM Manoussakis MN. Salivary gland epithelial cell exosomes: A source of autoantigenic ribonucleoproteins. Arthritis Rheum. (2005) 52:1517–21. doi: 10.1002/art.21005 15880835

[23] Ohlsson M Jonsson R Brokstad KA. Subcellular redistribution and surface exposure of the Ro52, Ro60 and La48 autoantigens during apoptosis in human ductal epithelial cells: a possible mechanism in the pathogenesis of Sjögren’s syndrome. Scand J Immunol. (2002) 56:456–69. doi: 10.1046/j.1365-3083.2002.01072_79.x 12410795

[24] Zhou J Pathak JL Liu Q Hu S Cao T Watanabe N. Modes and mechanisms of salivary gland epithelial cell death in sjogren’s syndrome. Adv Biol (Weinh). (2023) 7:e2300173. doi: 10.1002/adbi.202300173 37409392

[25] Shang YF Shen YY Zhang MC Lv MC Wang TY Chen XQ. Progress in salivary glands: Endocrine glands with immune functions. Front Endocrinol (Lausanne). (2023) 14:1061235. doi: 10.3389/fendo.2023.1061235 36817607 PMC9935576

[26] Porcheri C Mitsiadis TA. Physiology, pathology and regeneration of salivary glands. Cells. (2019) 8:976. doi: 10.3390/cells8090976 31455013 PMC6769486

[27] Xiang N Xu H Zhou Z Wang J Cai P Wang L. Single-cell transcriptome profiling reveals immune and stromal cell heterogeneity in primary Sjögren’s syndrome. iScience. (2023) 26:107943. doi: 10.1016/j.isci.2023.107943 37810210 PMC10558796

[28] Aure MH Symonds JM Mays JW Hoffman MP. Epithelial cell lineage and signaling in murine salivary glands. J Dent Res. (2019) 98:1186–94. doi: 10.1177/0022034519864592 PMC6755719 31331226

[29] Chibly AM Aure MH Patel VN Hoffman MP. Salivary gland function, development, and regeneration. Physiol Rev. (2022) 102:1495–552. doi: 10.1152/physrev.00015.2021 PMC9126227 35343828

[30] Emmerson E May AJ Berthoin L Cruz-Pacheco N Nathan S Mattingly AJ. Salivary glands regenerate after radiation injury through SOX2-mediated secretory cell replacement. EMBO Mol Med. (2018) 10:e8051. doi: 10.15252/emmm.201708051 29335337 PMC5840548

[31] Rugel-Stahl A Elliott ME Ovitt CE. Ascl3 marks adult progenitor cells of the mouse salivary gland. Stem Cell Res. (2012) 8:379–87. doi: 10.1016/j.scr.2012.01.002 PMC3319487 22370009

[32] Sneyd J Vera-Sigüenza E Rugis J Pages N Yule DI. Calcium dynamics and water transport in salivary acinar cells. Bull Math Biol. (2021) 83:31. doi: 10.1007/s11538-020-00841-9 33594615 PMC8018713

[33] Triantafyllou A Mikkelsen LH Gnepp DR Andreasen S Hunt JL Devaney KO. Salivary myoepithelial cells: an addendum. Ultrastruct Pathol. (2018) 42:465–76. doi: 10.1080/01913123.2018.1551259 30526219

[34] Su S Rugis J Wahl A Doak S Li Y Suresh V. A mathematical model of salivary gland duct cells. Bull Math Biol. (2022) 84:84. doi: 10.1007/s11538-022-01041-3 35799078 PMC9262821

[35] Wahl AM Takano T Su S Warner BM Perez P Sneyd J. Structural and functional analysis of salivary intercalated duct cells reveals a secretory phenotype. J Physiol. (2023) 601:4539–56. doi: 10.1113/JP285104 PMC10591963 37724716

[36] Carpenter GH. The secretion, components, and properties of saliva. Annu Rev Food Sci Technol. (2013) 4:267–76. doi: 10.1146/annurev-food-030212-182700 23464573

[37] Rodrigues Neves C Buskermolen J Roffel S Waaijman T Thon M Veerman E. Human saliva stimulates skin and oral wound healing in vitro. J Tissue Eng Regener Med. (2019) 13:1079–92. doi: 10.1002/term.2865 PMC6593997 30968584

[38] Dimitriou ID Kapsogeorgou EK Abu-Helu RF Moutsopoulos HM Manoussakis MN. Establishment of a convenient system for the long-term culture and study of non-neoplastic human salivary gland epithelial cells. Eur J Oral Sci. (2002) 110:21–30. doi: 10.1034/j.1600-0722.2002.00152.x 11878756

[39] van Ginkel MS Nakshbandi U Arends S Haacke EA Liefers SC Verstappen GM. Increased diagnostic accuracy of the labial gland biopsy in primary sjögren syndrome when multiple histopathological features are included. Arthritis Rheumatol. (2024) 76:421–8. doi: 10.1002/art.42723 37791984

[40] Asam S Neag G Berardicurti O Gardner D Barone F. The role of stroma and epithelial cells in primary Sjögren's syndrome. Rheumatol (Oxford). (2021) 60:3503–12. doi: 10.1093/rheumatology/kez050 30945742

[41] Low HZ Witte T. Aspects of innate immunity in Sjögren's syndrome. Arthritis Res Ther. (2011) 13:218. doi: 10.1186/ar3318 21635716 PMC3218872

[42] Kyriakidis NC Kapsogeorgou EK Gourzi VC Konsta OD Baltatzis GE Tzioufas AG. Toll-like receptor 3 stimulation promotes Ro52/TRIM21 synthesis and nuclear redistribution in salivary gland epithelial cells, partially via type I interferon pathway. Clin Exp Immunol. (2014) 178:548–60. doi: 10.1111/cei.12432 PMC4238881 25098814

[43] Ittah M Miceli-Richard C Gottenberg JE Sellam J Eid P Lebon P. Viruses induce high expression of BAFF by salivary gland epithelial cells through TLR- and type-I IFN-dependent and -independent pathways. Eur J Immunol. (2008) 38:1058–64. doi: 10.1002/eji.200738013 18350548

[44] Manoussakis MN Spachidou MP Maratheftis CI. Salivary epithelial cells from Sjogren’s syndrome patients are highly sensitive to anoikis induced by TLR-3 ligation. J Autoimmun. (2010) 35:212–8. doi: 10.1016/j.jaut.2010.06.010 20685081

[45] Horai Y Nakamura H Nakashima Y Hayashi T Kawakami A. Analysis of the downstream mediators of toll-like receptor 3-induced apoptosis in labial salivary glands in patients with Sjögren’s syndrome. Mod Rheumatol. (2016) 26:99–104. doi: 10.3109/14397595.2015.1045256 25926385

[46] Mingueneau M Boudaoud S Haskett S Reynolds TL Nocturne G Norton E. Cytometry by time-of-flight immunophenotyping identifies a blood Sjögren’s signature correlating with disease activity and glandular inflammation. J Allergy Clin Immunol. (2016) 137:1809–1821.e1812. doi: 10.1016/j.jaci.2016.01.024 27045581

[47] Rivière E Pascaud J Virone A Dupré A Ly B Paoletti A. Interleukin-7/interferon axis drives T cell and salivary gland epithelial cell interactions in sjögren’s syndrome. Arthritis Rheumatol. (2021) 73:631–40. doi: 10.1002/art.41558 33058491

[48] Ittah M Miceli-Richard C Eric Gottenberg J Lavie F Lazure T Ba N. B cell-activating factor of the tumor necrosis factor family (BAFF) is expressed under stimulation by interferon in salivary gland epithelial cells in primary Sjögren’s syndrome. Arthritis Res Ther. (2006) 8:R51. doi: 10.1186/ar1912 16507175 PMC1526588

[49] Waterman SA Gordon TP Rischmueller M. Inhibitory effects of muscarinic receptor autoantibodies on parasympathetic neurotransmission in Sjögren's syndrome. Arthritis Rheum. (2000) 43:1647–54. doi: 10.1002/(ISSN)1529-0131 10902771

[50] Teos LY Zhang Y Cotrim AP Swaim W Won JH Ambrus J. IP3R deficit underlies loss of salivary fluid secretion in Sjögren’s Syndrome. Sci Rep. (2015) 5:13953. doi: 10.1038/srep13953 26365984 PMC4568516

[51] Cortés J Hidalgo J Aguilera S Castro I Brito M Urra H. Synaptotagmin-1 overexpression under inflammatory conditions affects secretion in salivary glands from Sjögren’s syndrome patients. J Autoimmun. (2019) 97:88–99. doi: 10.1016/j.jaut.2018.10.019 30391023

[52] Saito E Watari I Mizumachi-Kubono M Hsu-Hayashi S Ono T. Occlusional modifications reversibly alter aquaporin 5 expression and localization in rat salivary glands. Front Physiol. (2020) 11:528. doi: 10.3389/fphys.2020.00528 32587522 PMC7298139

[53] Sung HH Castro I González S Aguilera S Smorodinsky NI Quest A. MUC1/SEC and MUC1/Y overexpression is associated with inflammation in Sjögren’s syndrome. Oral Dis. (2015) 21:730–8. doi: 10.1111/odi.12339 25757505

[54] Castro I Albornoz N Aguilera S Barrera MJ González S Núñez M. Aberrant MUC1 accumulation in salivary glands of Sjögren’s syndrome patients is reversed by TUDCA in vitro. Rheumatol (Oxford). (2020) 59:742–53. doi: 10.1093/rheumatology/kez316 31377809

[55] Herrera-Esparza R Bollain-y-Goytia J Ruvalcaba C Ruvalcaba M Pacheco-Tovar D Avalos-Díaz E. Apoptosis and cell proliferation: the paradox of salivary glands in Sjögren’s disease. Acta Reumatol Port. (2008) 33:299–303. 18846009

[56] Matsumura R Umemiya K Goto T Nakazawa T Ochiai K Kagami M. Interferon gamma and tumor necrosis factor alpha induce Fas expression and anti-Fas mediated apoptosis in a salivary ductal cell line. Clin Exp Rheumatol. (2000) 18:311–8. 10895367

[57] Kong L Ogawa N McGuff HS Nakabayashi T Sakata KM Masago R. Bcl-2 family expression in salivary glands from patients with primary Sjögren’s syndrome: involvement of Bax in salivary gland destruction. Clin Immunol Immunopathol. (1998) 88:133–41. doi: 10.1006/clin.1998.4556 9714690

[58] Molina C Alliende C Aguilera S Kwon YJ Leyton L Martínez B. Basal lamina disorganisation of the acini and ducts of labial salivary glands from patients with Sjogren’s syndrome: association with mononuclear cell infiltration. Ann Rheum Dis. (2006) 65:178–83. doi: 10.1136/ard.2004.033837 PMC1798011 16014676

[59] van Ginkel MS Haacke EA Bootsma H Arends S van Nimwegen JF Verstappen GM. Presence of intraepithelial B-lymphocytes is associated with the formation of lymphoepithelial lesions in salivary glands of primary Sjögren’s syndrome patients. Clin Exp Rheumatol. (2019) 37 Suppl 118:42–8. 31074726

[60] Mauro D Lin X Pontarini E Wehr P Guggino G Tang Y. CD8(+) tissue-resident memory T cells are expanded in primary Sjögren’s disease and can be therapeutically targeted by CD103 blockade. Ann Rheum Dis. (2024) 225069. doi: 10.1136/ard-2023-225069 38777379

[61] Xu T Zhu HX You X Ma JF Li X Luo PY. Single-cell profiling reveals pathogenic role and differentiation trajectory of granzyme K+CD8+ T cells in primary Sjögren’s syndrome. JCI Insight. (2023) 8:e167490. doi: 10.1172/jci.insight.167490 36881472 PMC10243796

[62] Blokland SLM Flessa CM van Roon JAG Mavragani CP. Emerging roles for chemokines and cytokines as orchestrators of immunopathology in Sjögren’s syndrome. Rheumatol (Oxford). (2021) 60:3072–87. doi: 10.1093/rheumatology/key438 30838419

[63] Lämmermann T Kastenmüller W. Concepts of GPCR-controlled navigation in the immune system. Immunol Rev. (2019) 289:205–31. doi: 10.1111/imr.12752 PMC6487968 30977203

[64] Bachelerie F Ben-Baruch A Burkhardt AM Combadiere C Farber JM Graham GJ. International Union of Basic and Clinical Pharmacology. [corrected]. LXXXIX. Update on the extended family of chemokine receptors and introducing a new nomenclature for atypical chemokine receptors. Pharmacol Rev. (2014) 66:1–79. doi: 10.1124/pr.113.007724 24218476 PMC3880466

[65] Mempel TR Lill JK Altenburger LM. How chemokines organize the tumour microenvironment. Nat Rev Cancer. (2024) 24:28–50. doi: 10.1038/s41568-023-00635-w 38066335 PMC11480775

[66] Cuello C Palladinetti P Tedla N Di Girolamo N Lloyd AR McCluskey PJ. Chemokine expression and leucocyte infiltration in Sjögren’s syndrome. Br J Rheumatol. (1998) 37:779–83. doi: 10.1093/rheumatology/37.7.779 9714357

[67] Barone F Bombardieri M Rosado MM Morgan PR Challacombe SJ De Vita S. CXCL13, CCL21, and CXCL12 expression in salivary glands of patients with Sjogren’s syndrome and MALT lymphoma: association with reactive and Malignant areas of lymphoid organization. J Immunol. (2008) 180:5130–40. doi: 10.4049/jimmunol.180.7.5130 18354239

[68] Sfriso P Oliviero F Calabrese F Miorin M Facco M Contri A. Epithelial CXCR3-B regulates chemokines bioavailability in normal, but not in Sjogren’s syndrome, salivary glands. J Immunol. (2006) 176:2581–9. doi: 10.4049/jimmunol.176.4.2581 16456020

[69] Zhao L Xu W Chen Z Zhang H Zhang S Lian C. Aberrant distribution of CD3+CD56+ NKT-like cells in patients with primary Sjögren’s syndrome. Clin Exp Rheumatol. (2021) 39:98–104. doi: 10.55563/clinexprheumatol/uzzz6d 32242817

[70] Tsubota K Nishiyama T Mishima K Inoue H Doi T Hattori Y. The role of fractalkine as an accelerating factor on the autoimmune exocrinopathy in mice. Invest Ophthalmol Vis Sci. (2009) 50:4753–60. doi: 10.1167/iovs.08-2596 19407023

[71] Hernandez-Segura A Nehme J Demaria M. Hallmarks of cellular senescence. Trends Cell Biol. (2018) 28:436–53. doi: 10.1016/j.tcb.2018.02.001 29477613

[72] Hughes CE Nibbs RJB. A guide to chemokines and their receptors. FEBS J. (2018) 285:2944–71. doi: 10.1111/febs.14466 PMC6120486 29637711

[73] David BA Kubes P. Exploring the complex role of chemokines and chemoattractants in *vivo* on leukocyte dynamics. Immunol Rev. (2019) 289:9–30. doi: 10.1111/imr.12757 30977202

[74] Blokland SLM Hillen MR Kruize AA Meller S Homey B Smithson GM. Increased CCL25 and T helper cells expressing CCR9 in the salivary glands of patients with primary sjögren’s syndrome: potential new axis in lymphoid neogenesis. Arthritis Rheumatol. (2017) 69:2038–51. doi: 10.1002/art.40182 28622456

[75] Blokland SLM Kislat A Homey B Smithson GM Kruize AA Radstake T. Decreased circulating CXCR3 + CCR9+T helper cells are associated with elevated levels of their ligands CXCL10 and CCL25 in the salivary gland of patients with Sjögren’s syndrome to facilitate their concerted migration. Scand J Immunol. (2020) 91:e12852. doi: 10.1111/sji.12852 31733111 PMC7064901

[76] Papadakis KA Prehn J Moreno ST Cheng L Kouroumalis EA Deem R. CCR9-positive lymphocytes and thymus-expressed chemokine distinguish small bowel from colonic Crohn’s disease. Gastroenterology. (2001) 121:246–54. doi: 10.1053/gast.2001.27154 11487533

[77] Ogawa N Ping L Zhenjun L Takada Y Sugai S. Involvement of the interferon-gamma-induced T cell-attracting chemokines, interferon-gamma-inducible 10-kd protein (CXCL10) and monokine induced by interferon-gamma (CXCL9), in the salivary gland lesions of patients with Sjögren’s syndrome. Arthritis Rheum. (2002) 46:2730–41. doi: 10.1002/art.10577 12384933

[78] Vakrakou AG Polyzos A Kapsogeorgou EK Thanos D Manoussakis MN. Impaired anti-inflammatory activity of PPARγ in the salivary epithelia of Sjögren’s syndrome patients imposed by intrinsic NF-κB activation. J Autoimmun. (2018) 86:62–74. doi: 10.1016/j.jaut.2017.09.007 29033144

[79] Guggino G Lin X Rizzo A Xiao F Saieva L Raimondo S. Interleukin-25 axis is involved in the pathogenesis of human primary and experimental murine sjögren’s syndrome. Arthritis Rheumatol. (2018) 70:1265–75. doi: 10.1002/art.40500 29569854

[80] Sakai A Sugawara Y Kuroishi T Sasano T Sugawara S. Identification of IL-18 and Th17 cells in salivary glands of patients with Sjögren’s syndrome, and amplification of IL-17-mediated secretion of inflammatory cytokines from salivary gland cells by IL-18. J Immunol. (2008) 181:2898–906. doi: 10.4049/jimmunol.181.4.2898 18684981

[81] Nakamura H Shimizu T Kawakami A. Role of viral infections in the pathogenesis of sjögren’s syndrome: different characteristics of epstein-barr virus and HTLV-1. J Clin Med. (2020) 9:1459. doi: 10.3390/jcm9051459 32414149 PMC7290771

[82] Borza CM Hutt-Fletcher LM. Alternate replication in B cells and epithelial cells switches tropism of Epstein-Barr virus. Nat Med. (2002) 8:594–9. doi: 10.1038/nm0602-594 12042810

[83] Inoue H Mishima K Yamamoto-Yoshida S Ushikoshi-Nakayama R Nakagawa Y Yamamoto K. Aryl hydrocarbon receptor-mediated induction of EBV reactivation as a risk factor for Sjögren’s syndrome. J Immunol. (2012) 188:4654–62. doi: 10.4049/jimmunol.1101575 22467650

[84] Vakrakou AG Svolaki IP Evangelou K Gorgoulis VG Manoussakis MN. Cell-autonomous epithelial activation of AIM2 (absent in melanoma-2) inflammasome by cytoplasmic DNA accumulations in primary Sjögren’s syndrome. J Autoimmun. (2020) 108:102381. doi: 10.1016/j.jaut.2019.102381 31919014

[85] Fox PC Brennan M Di Sun P. Cytokine expression in human labial minor salivary gland epithelial cells in health and disease. Arch Oral Biol. (1999) 44 Suppl 1:S49–52. doi: 10.1016/S0003-9969(99)90018-3 10414856

[86] Apostolou E Kapsogeorgou EK Konsta OD Giotakis I Saridaki MI Andreakos E. Expression of type III interferons (IFNλs) and their receptor in Sjögren’s syndrome. Clin Exp Immunol. (2016) 186:304–12. doi: 10.1111/cei.12865 PMC5108072 27613139

[87] Ha YJ Choi YS Kang EH Chung JH Cha S Song YW. Increased expression of interferon-λ in minor salivary glands of patients with primary Sjögren’s syndrome and its synergic effect with interferon-α on salivary gland epithelial cells. Clin Exp Rheumatol. (2018) 36 Suppl 112:31–40. 28421993

[88] Nayar S Campos J Smith CG Iannizzotto V Gardner DH Mourcin F. Immunofibroblasts are pivotal drivers of tertiary lymphoid structure formation and local pathology. Proc Natl Acad Sci U.S.A. (2019) 116:13490–7. doi: 10.1073/pnas.1905301116 PMC6613169 31213547

[89] Sisto M Lorusso L Lisi S. TLR2 signals via NF-κB to drive IL-15 production in salivary gland epithelial cells derived from patients with primary Sjögren’s syndrome. Clin Exp Med. (2017) 17:341–50. doi: 10.1007/s10238-016-0429-y 27260411

[90] Liu X Wang H Wang X Jiang X Jin Y Han Y. Identification and verification of inflammatory biomarkers for primary Sjögren’s syndrome. Clin Rheumatol. (2024) 43:1335–52. doi: 10.1007/s10067-024-06901-y PMC10944815 38376769

[91] Becher B Tugues S Greter M. GM-CSF: from growth factor to central mediator of tissue inflammation. Immunity. (2016) 45:963–73. doi: 10.1016/j.immuni.2016.10.026 27851925

[92] Fink M Wrana JL. Regulation of homeostasis and regeneration in the adult intestinal epithelium by the TGF-β superfamily. Dev Dyn. (2023) 252:445–62. doi: 10.1002/dvdy.500 35611490

[93] Sun D Emmert-Buck MR Fox PC. Differential cytokine mRNA expression in human labial minor salivary glands in primary Sjögren’s syndrome. Autoimmunity. (1998) 28:125–37. doi: 10.3109/08916939808996281 9867125

[94] Zhang X Yun JS Han D Yook JI Kim HS Cho ES. TGF-β Pathway in salivary gland fibrosis. Int J Mol Sci. (2020) 21:9138. doi: 10.3390/ijms21239138 33266300 PMC7730716

[95] Travis MA Sheppard D. TGF-β activation and function in immunity. Annu Rev Immunol. (2014) 32:51–82. doi: 10.1146/annurev-immunol-032713-120257 24313777 PMC4010192

[96] Ebina-Shibuya R Leonard WJ. Role of thymic stromal lymphopoietin in allergy and beyond. Nat Rev Immunol. (2023) 23:24–37. doi: 10.1038/s41577-022-00735-y 35650271 PMC9157039

[97] Hillen MR Kruize AA Bikker A Wenting-van Wijk M Radstake TR Hack CE. Decreased expression of thymic stromal lymphopoietin in salivary glands of patients with primary Sjögren’s syndrome is associated with increased disease activity. Mod Rheumatol. (2016) 26:105–9. doi: 10.3109/14397595.2015.1054089 25995032

[98] Conti P Stellin L Caraffa A Gallenga CE Ross R Kritas SK. Advances in mast cell activation by IL-1 and IL-33 in sjögren's syndrome: promising inhibitory effect of IL-37. Int J Mol Sci. (2020) 21:4297. doi: 10.3390/ijms21124297 32560266 PMC7352728

[99] Apostolou E Tzioufas AG. Type-III interferons in Sjögren’s syndrome. Clin Exp Rheumatol. (2020) 38 Suppl 126:245–52. 32896259

[100] Groom J Kalled SL Cutler AH Olson C Woodcock SA Schneider P. Association of BAFF/BLyS overexpression and altered B cell differentiation with Sjögren’s syndrome. J Clin Invest. (2002) 109:59–68. doi: 10.1172/JCI0214121 11781351 PMC150825

[101] Jonsson MV Szodoray P Jellestad S Jonsson R Skarstein K. Association between circulating levels of the novel TNF family members APRIL and BAFF and lymphoid organization in primary Sjögren’s syndrome. J Clin Immunol. (2005) 25:189–201. doi: 10.1007/s10875-005-4091-5 15981083

[102] Nocturne G Mariette X. B cells in the pathogenesis of primary Sjögren syndrome. Nat Rev Rheumatol. (2018) 14:133–45. doi: 10.1038/nrrheum.2018.1 29416129

[103] Hansen A Daridon C Dörner T. What do we know about memory B cells in primary Sjögren's syndrome? Autoimmun Rev. (2010) 9:600–3. doi: 10.1016/j.autrev.2010.05.005 20452465

[104] Corfe SA Paige CJ. The many roles of IL-7 in B cell development; mediator of survival, proliferation and differentiation. Semin Immunol. (2012) 24:198–208. doi: 10.1016/j.smim.2012.02.001 22421572

[105] Carrette F Surh CD. IL-7 signaling and CD127 receptor regulation in the control of T cell homeostasis. Semin Immunol. (2012) 24:209–17. doi: 10.1016/j.smim.2012.04.010 PMC3367861 22551764

[106] Jin JO Kawai T Cha S Yu Q. Interleukin-7 enhances the Th1 response to promote the development of Sjögren’s syndrome-like autoimmune exocrinopathy in mice. Arthritis Rheum. (2013) 65:2132–42. doi: 10.1002/art.38007 PMC3729733 23666710

[107] Monteith AJ Kang S Scott E Hillman K Rajfur Z Jacobson K. Defects in lysosomal maturation facilitate the activation of innate sensors in systemic lupus erythematosus. Proc Natl Acad Sci U.S.A. (2016) 113:E2142–2151. doi: 10.1073/pnas.1513943113 PMC4839468 27035940

[108] Ware MB Wolfarth AA Goon JB Ezeanya UI Dhar S Ferrando-Martinez S. The role of interleukin-7 in the formation of tertiary lymphoid structures and their prognostic value in gastrointestinal cancers. J Immunother Precis Oncol. (2022) 5:105–17. doi: 10.36401/JIPO-22-10 PMC9714415 36483588

[109] Sarrand J Baglione L Parisis D Soyfoo M. The involvement of alarmins in the pathogenesis of sjögren’s syndrome. Int J Mol Sci. (2022) 23:5671. doi: 10.3390/ijms23105671 35628481 PMC9145074

[110] Nakamura H Horai Y Shimizu T Kawakami A. Modulation of apoptosis by cytotoxic mediators and cell-survival molecules in sjögren’s syndrome. Int J Mol Sci. (2018) 19:2369. doi: 10.3390/ijms19082369 30103522 PMC6121505

[111] Ainola M Porola P Takakubo Y Przybyla B Kouri VP Tolvanen TA. Activation of plasmacytoid dendritic cells by apoptotic particles - mechanism for the loss of immunological tolerance in Sjögren’s syndrome. Clin Exp Immunol. (2018) 191:301–10. doi: 10.1111/cei.13077 PMC5801512 29105068

[112] Tanaka T Warner BM Odani T Ji Y Mo YQ Nakamura H. LAMP3 induces apoptosis and autoantigen release in Sjögren’s syndrome patients. Sci Rep. (2020) 10:15169. doi: 10.1038/s41598-020-71669-5 32939030 PMC7494869

[113] ColaFrancesco S Barbati C Priori R Putro E Giardina F Gattamelata A. Maladaptive autophagy in the pathogenesis of autoimmune epithelitis in sjögren’s syndrome. Arthritis Rheumatol. (2022) 74:654–64. doi: 10.1002/art.42018 34748286

[114] Li B Wang F Schall N Muller S. Rescue of autophagy and lysosome defects in salivary glands of MRL/lpr mice by a therapeutic phosphopeptide. J Autoimmun. (2018) 90:132–45. doi: 10.1016/j.jaut.2018.02.005 29486915

[115] Hong SM Lee J Jang SG Lee J Cho ML Kwok SK. Type I interferon increases inflammasomes associated pyroptosis in the salivary glands of patients with primary sjögren’s syndrome. Immune Netw. (2020) 20:e39. doi: 10.4110/in.2020.20.e39 33163247 PMC7609163

[116] Hwang SH Woo JS Moon J Yang S Park JS Lee J. IL-17 and CCR9(+)α4β7(-) th17 cells promote salivary gland inflammation, dysfunction, and cell death in sjögren’s syndrome. Front Immunol. (2021) 12:721453. doi: 10.3389/fimmu.2021.721453 34539657 PMC8440850

[117] Cao T Zhou J Liu Q Mao T Chen B Wu Q. Interferon-γ induces salivary gland epithelial cell ferroptosis in Sjogren’s syndrome via JAK/STAT1-mediated inhibition of system Xc(). Free Radic Biol Med. (2023) 205:116–28. doi: 10.1016/j.freeradbiomed.2023.05.027 37286044

[118] Zhou J Pathak JL Wu L Chen B Cao T Wei W. Downregulated GPX4 in salivary gland epithelial cells contributes to salivary secretion dysfunction in Sjogren’s syndrome via lipid ROS/pSTAT4/AQP5 axis. Free Radic Biol Med. (2024) 218:1–15. doi: 10.1016/j.freeradbiomed.2024.04.003 38574973

[119] Dong Y Ming B Dong L. The role of HMGB1 in rheumatic diseases. Front Immunol. (2022) 13:815257. doi: 10.3389/fimmu.2022.815257 35250993 PMC8892237

[120] Lu B Antoine DJ Kwan K Lundbäck P Wähämaa H Schierbeck H. JAK/STAT1 signaling promotes HMGB1 hyperacetylation and nuclear translocation. Proc Natl Acad Sci U.S.A. (2014) 111:3068–73. doi: 10.1073/pnas.1316925111 PMC3939889 24469805

[121] Hou L Yang Z Wang Z Zhang X Zhao Y Yang H. NLRP3/ASC-mediated alveolar macrophage pyroptosis enhances HMGB1 secretion in acute lung injury induced by cardiopulmonary bypass. Lab Invest. (2018) 98:1052–64. doi: 10.1038/s41374-018-0073-0 29884910

[122] Li W Deng M Loughran PA Yang M Lin M Yang C. LPS induces active HMGB1 release from hepatocytes into exosomes through the coordinated activities of TLR4 and caspase-11/GSDMD signaling. Front Immunol. (2020) 11:229. doi: 10.3389/fimmu.2020.00229 32328059 PMC7160675

[123] Kanne AM Jülich M Mahmutovic A Tröster I Sehnert B Urbonaviciute V. Association of high mobility group box chromosomal protein 1 and receptor for advanced glycation end products serum concentrations with extraglandular involvement and disease activity in sjögren’s syndrome. Arthritis Care Res (Hoboken). (2018) 70:944–8. doi: 10.1002/acr.23420 28941024

[124] Wang D Zhou M Wang Y Sun S. Suppression of high-mobility group box 1 ameliorates xerostomia in a Sjögren syndrome-triggered mouse model. Can J Physiol Pharmacol. (2020) 98:351–6. doi: 10.1139/cjpp-2019-0337 31935120

[125] Cavalli G ColaFrancesco S Emmi G Imazio M Lopalco G Maggio MC. Interleukin 1α: a comprehensive review on the role of IL-1α in the pathogenesis and treatment of autoimmune and inflammatory diseases. Autoimmun Rev. (2021) 20:102763. doi: 10.1016/j.autrev.2021.102763 33482337

[126] He PY Wu MY Zheng LY Duan Y Fan Q Zhu XM. Interleukin-33/serum stimulation-2 pathway: Regulatory mechanisms and emerging implications in immune and inflammatory diseases. Cytokine Growth Factor Rev. (2024) 76:112–26. doi: 10.1016/j.cytogfr.2023.12.001 38155038

[127] Brunner TM Serve S Marx AF Fadejeva J Saikali P Dzamukova M. A type 1 immunity-restricted promoter of the IL-33 receptor gene directs antiviral T-cell responses. Nat Immunol. (2024) 25:256–67. doi: 10.1038/s41590-023-01697-6 PMC10834369 38172258

[128] Awada A Nicaise C Ena S Schandéné L Rasschaert J Popescu I. Potential involvement of the IL-33-ST2 axis in the pathogenesis of primary Sjogren’s syndrome. Ann Rheum Dis. (2014) 73:1259–63. doi: 10.1136/annrheumdis-2012-203187 24385203

[129] Bowman SJ Fox R Dörner T Mariette X Papas A Grader-Beck T. Safety and efficacy of subcutaneous ianalumab (VAY736) in patients with primary Sjögren’s syndrome: a randomised, double-blind, placebo-controlled, phase 2b dose-finding trial. Lancet. (2022) 399:161–71. doi: 10.1016/S0140-6736(21)02251-0 34861168

[130] Bai W Liu H Dou L Yang Y Leng X Li M. Pilot study of baricitinib for active Sjogren’s syndrome. Ann Rheum Dis. (2022) 81:1050–2. doi: 10.1136/annrheumdis-2021-222053 35338034

[131] Xu D Fang J Zhang S Huang C Huang C Qin L. Efficacy and safety of telitacicept in primary Sjögren’s syndrome: a randomized, double-blind, placebo-controlled, phase 2 trial. Rheumatol (Oxford). (2024) 63:698–705. doi: 10.1093/rheumatology/kead265 37399108

[132] Pringle S Wang X Bootsma H Spijkervet FKL Vissink A Kroese FGM. Small-molecule inhibitors and the salivary gland epithelium in Sjögren’s syndrome. Expert Opin Investig Drugs. (2019) 28:605–16. doi: 10.1080/13543784.2019.1631796 31203680

[133] Amor C Feucht J Leibold J Ho YJ Zhu C Alonso-Curbelo D. Senolytic CAR T cells reverse senescence-associated pathologies. Nature. (2020) 583:127–32. doi: 10.1038/s41586-020-2403-9 PMC7583560 32555459

[134] Feng J van der Zwaag M Stokman MA van Os R Coppes RP. Isolation and characterization of human salivary gland cells for stem cell transplantation to reduce radiation-induced hyposalivation. Radiother Oncol. (2009) 92:466–71. doi: 10.1016/j.radonc.2009.06.023 19625095

[135] Lombaert IM Brunsting JF Wierenga PK Faber H Stokman MA Kok T. Rescue of salivary gland function after stem cell transplantation in irradiated glands. PloS One. (2008) 3:e2063. doi: 10.1371/journal.pone.0002063 18446241 PMC2329592

